# Management of varices but not anticoagulation is associated with improved outcome in patients with HCC and macrovascular tumour invasion

**DOI:** 10.1186/s40644-024-00657-z

**Published:** 2024-01-13

**Authors:** Lorenz Balcar, Arpad Mrekva, Bernhard Scheiner, Katharina Pomej, Tobias Meischl, Mattias Mandorfer, Thomas Reiberger, Michael Trauner, Dietmar Tamandl, Matthias Pinter

**Affiliations:** 1 Division of Gastroenterology and Hepatology, Department of Medicine III, Medical University of Vienna, Waehringer Guertel 18-20, Vienna, 1090 Austria; 2 Liver Cancer (HCC) Study Group Vienna, Medical University of Vienna, Vienna, Austria; 3 Department of Biomedical Imaging and Image-Guided Therapy, Medical University of Vienna, Vienna, Austria; 4 3rd Medical Department (Hematology & Oncology), Hanusch Krankenhaus, Vienna, Austria

**Keywords:** Liver cancer, Beta blockers, Variceal bleeding, Portal hypertension, Varices

## Abstract

**Background & aims:**

The value of bleeding prophylaxis and anticoagulation in patients with hepatocellular carcinoma (HCC) and macrovascular tumour invasion (MVI) is unclear. We evaluated the impact of anticoagulation on thrombosis progression, bleeding events, and overall mortality, and assessed the efficacy of adequate management of varices as recommended for patients with cirrhosis.

**Methods:**

HCC patients with MVI who had Child-Turcotte-Pugh A-B7 were included between Q4/2002 and Q2/2022. Localization of the tumour thrombus and changes at 3–6 months were evaluated by two radiologists. Univariable and multivariable logistic/Cox regression analyses included time-dependent variables (i.e., anticoagulation, systemic therapy, non-selective beta blocker treatment).

**Results:**

Of 124 patients included (male: *n* = 110, 89%), MVI involved the main portal vein in 47 patients (38%), and 49 individuals (40%) had additional non-tumorous thrombus apposition. Fifty of 80 patients (63%) with available endoscopy had varices. Twenty-four individuals (19%) received therapeutic anticoagulation and 94 patients (76%) were treated with effective systemic therapies. The use of therapeutic anticoagulation did not significantly affect the course of the malignant thrombosis at 3–6 months. Systemic therapy (aHR: 0.26 [95%CI: 0.16–0.40]) but not anticoagulation was independently associated with reduced all-cause mortality. In patients with known variceal status, adequate management of varices was independently associated with reduced risk of variceal bleeding (aHR: 0.12 [95%CI: 0.02–0.71]). In the whole cohort, non-selective beta blockers were independently associated with reduced risk of variceal bleeding or death from any cause (aHR: 0.69 [95%CI: 0.50–0.96]).

**Conclusion:**

Adequate bleeding prophylaxis and systemic anti-tumour therapy but not anticoagulation were associated with improved outcomes in patients with HCC and MVI.

**Supplementary Information:**

The online version contains supplementary material available at 10.1186/s40644-024-00657-z.

## Introduction

Hepatocellular carcinoma (HCC) is the most common primary liver cancer and usually develops in patients with liver cirrhosis or an underlying chronic liver disease [1–3]. Macrovascular tumour invasion into portal or hepatic veins is a common complication of advanced or progressing HCC and associated with a poor prognosis [4–6]. Given the critical implications for management, portal vein tumour thrombosis needs to be distinguished from non-tumorous portal vein thrombosis – a well-known complication in patients with liver cirrhosis [7]. This can be done by contrast-enhanced imaging with relatively high accuracy [8, 9]. Patients with HCC who developed macrovascular tumour invasion become candidates for systemic therapy. While immune checkpoint inhibitor (ICI)-based combination therapies have replaced tyrosine kinase inhibitors (TKIs) as preferred standard of care in systemic front-line, TKIs are frequently used in the second-line setting [10].

Macrovascular tumour invasion, particularly in case of main portal vein involvement, is one of the most important negative prognostic factors [11] and may complicate the course of the disease by aggravating portal hypertension and its complications [12]. Indeed, portal vein tumour thrombosis is associated with an increased risk of high-risk varices and variceal bleeding in patients with HCC [13]. Moreover, invasion of hepatic veins or the vena cava may increase the risk for venous thromboembolism [14, 15].

Current recommendations on the prevention of variceal bleeding in individuals with liver cirrhosis also apply to patients with cirrhosis and HCC [12, 16, 17]. However, the value of adequate bleeding prophylaxis, particularly that of non-selective beta blockers (NSBB), in patients with clinically significant portal hypertension secondary to portal vein tumour thrombosis is unclear [12].

Moreover, data on anticoagulation to prevent or treat non-malignant thrombus apposition in HCC patients with macrovascular tumour invasion are lacking. While therapeutic anticoagulation is recommended in selected patients with liver cirrhosis and non-tumorous portal vein thrombosis [7, 18], no recommendations exist for HCC patients with portal vein tumour thrombosis.

To address some of these knowledge gaps, we conducted a retrospective study in patients with advanced HCC and well-preserved liver function who were diagnosed with macrovascular tumour invasion. We investigated the impact of anticoagulation on thrombosis progression, bleeding events, and all-cause mortality, and assessed the efficacy of adequate management of varices as recommended for patients with cirrhosis.

## Methods

### Study design and patients

Patients with histologically or radiologically diagnosed HCC and suspected macrovascular tumour invasion were considered for this retrospective study. Since patients with decompensated liver disease should receive best supportive care [3], we only included patients with Child-Turcotte-Pugh (CTP) score A or B7. Individuals with Barcelona-Clinic Liver Cancer (BCLC) stage D or unconfirmed macrovascular tumour invasion (i.e., only non-tumorous thrombosis), as well as patients treated with surgery or locoregional therapies (i.e., transarterial chemoembolization [TACE], selective internal radiation therapy [SIRT], or ablation, as these therapies are not recommended for HCC with macrovascular tumour invasion [3]) were excluded. Furthermore, we excluded individuals who were lost to follow-up within 30 days. Only patients with available images of the scans (i.e., computed tomography [CT] or magnetic resonance imaging [MRI]) at diagnosis of macrovascular tumour invasion were eligible. Patients were included between Q4/2002 and Q2/2022 at the Medical University of Vienna/General Hospital Vienna. Data including patient history and laboratory results were collected retrospectively. The date of diagnosis of macrovascular tumour invasion was defined as the baseline of this study. The retrospective analysis was approved by the Ethics Committee of the Medical University of Vienna.

### Radiological assessments

Contrast-enhanced CT and/or MRI scans were performed at baseline and approximately every 3 months thereafter. Images were read in consensus by two radiologists (A.M., and D.T.) who were blinded regarding medical treatment. The following characteristics of the tumour thrombus were described at baseline: location and extension of macrovascular tumour invasion, grade of occlusion of affected vessel (total/partial), type of thrombus (tumour thrombus or non-tumorous thrombus apposition), and presence of venous congestion.

Differentiation between tumour and non-tumorous thrombus was performed according to the following criteria: According to the current Liver Imaging and Data System (LI-RADS), the presence of unequivocal enhancing soft tissue in a vein, regardless of visualisation of parenchymal mass was considered a feature diagnostic of tumorous thrombus. Further features indicating tumorous thrombus were occluded vein with restricted diffusion, occluded or obscured vein in contiguity with malignant parenchymal mass and heterogeneous vein appearance not attributable to an artifact, leading to further assessment whether an enhancing component in the thrombus was to be observed [19].

In patients with available follow-up imaging, changes in thrombus size or degree of occlusion were assessed by direct comparison with the last imaging performed immediately before, and evaluated according to Baveno VII recommendations [16]: i) regression – thrombus decreased in size or degree of occlusion; ii) stabilization – no appreciable change in size or occlusion; iii) progression – thrombus increased in size or degree of occlusion. Best radiological response of the thrombus was evaluated at 3–6 months, as recommended in patients with liver cirrhosis and portal vein thrombosis undergoing anticoagulation [7].

### Management of macrovascular tumour invasion and varices

Patient data was obtained from medical records. Start and stop date of anticoagulation and systemic anti-tumour therapy was recorded for time-dependent analyses. The following therapies were regarded as ‘effective systemic therapies’, as these are recommended in advanced HCC with macrovascular tumour invasion [20, 21]: immune checkpoint inhibitor (ICI)-based therapies, sorafenib, lenvatinib, regorafenib, cabozantinib, and ramucirumab. Non-effective treatment included any other systemic therapy (i.e., experimental or with unproven efficacy in HCC, including octreotide, sirolimus, crizotinib, thalidomide, nintedanib, tivantinib, imatinib), or no specific anti-tumour therapy.

The decision on whether to initiate anticoagulation as well as the type of anticoagulation were solely at the discretion of the treating physician, as international or local guidelines on anticoagulation in patients with HCC and macrovascular tumour invasion are lacking. For main analyses, anticoagulation was considered adequate if therapeutic doses were used, and only these were included in the ‘therapeutic anticoagulation’ group, whereas patients receiving reduced/prophylactic doses were excluded. However, for sensitivity analysis, we also calculated Cox regression models including patients who received any dose of anticoagulation.

Management of varices was evaluated in patients with known variceal status. Accordingly, the following clinical scenarios were considered as adequate management of varices [16, 22]: i) low-risk varices – no endoscopic treatment, NSBB optionally, ii) high-risk varices – either NSBB or endoscopic treatment (or both), and iii) history of variceal bleeding – NSBB plus endoscopic treatment.

Portal hypertension-related complications (i.e., variceal bleeding, ascites, hepatorenal syndrome-type acute kidney injury, spontaneous bacterial peritonitis, overt hepatic encephalopathy) during follow-up were obtained from medical records.

### Statistical analysis

Statistical analyses were performed using IBM SPSS Statistics version 27 (SPSS Inc., Chicago, IL), R 4.1.2 (R Core Team, R Foundation for Statistical Computing, Vienna, Austria) and GraphPad Prism 8 (GraphPad Software, Inc., San Diego, CA). As this is a retrospective study, no formal sample size calculation was performed, instead, all available patients fulfilling inclusion but not exclusion criteria were considered for this study. Data on baseline patient and tumour characteristics as well as radiographic features were summarised using descriptive statistics. Categorical variables were reported as absolute (n) and relative frequencies (%), whereas continuous variables as mean ± standard deviation (SD) or median (interquartile range [IQR]), as appropriate. Student’s t-test was used for group comparisons of normally distributed variables and Mann-Whitney-U-test for non-normally distributed variables. Group comparisons of categorical variables were performed using either Chi-squared or Fisher’s exact test, as appropriate. Logistic regression analyses were calculated with variceal bleeding as outcome of interest using backward elimination for variable selection in patients with known endoscopy status at study inclusion.

Overall survival was defined as the time from radiological diagnosis of macrovascular tumour invasion until death, and patients who were still alive were censored at the date of last contact. Variceal bleeding-free survival was defined as time from radiological diagnosis of macrovascular tumour invasion until variceal bleeding or death from any cause, whatever came first; patients who were still alive without variceal bleeding were censored at the date of last contact. Time on treatment (e.g., systemic anti-tumour therapy, anticoagulation, NSBB) was defined as the time from treatment start until end of treatment (e.g., including time on 1st and further lines of systemic anti-tumour therapy); patients who were alive or lost to follow-up with ongoing treatment were censored at the date of last contact. Median overall survival was calculated by the Kaplan-Meier method. Median estimated follow-up was calculated using the reverse Kaplan-Meier method [23].

Univariable and multivariable analyses were conducted using Cox regression analyses, and included time-dependent variables (i.e., anticoagulation, effective systemic therapy, NSBB; https://cran.r-project.org/web/packages/survival/vignettes/timedep.pdf). Data was put into long-format using the tmerge package (https://www.rdocumentation.org/packages/survival/versions/2.43-3/topics/tmerge). Variable selection was based on backward elimination, eliminating variables with *p*-values > 0.157 [24]. For graphical depiction, a Simon-Makuch plot was created [25, 26]. The Sankey plot was created using SankeyMATIC (https://sankeymatic.com). The level of significance was set at a 2-sided *p*-value < 0.05.

## Results

### Study population and patient characteristics

**Fig. 1:**
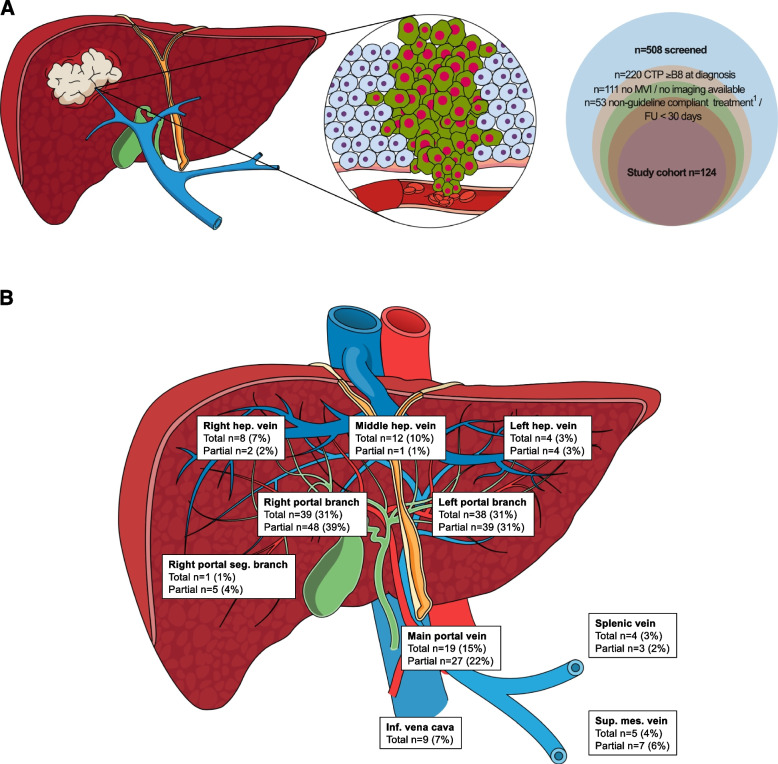
Patient flow chart and location of macrovascular tumour invasion. **A** A total of 508 patients diagnosed with hepatocellular carcinoma and suspected macrovascular tumour invasion between Q4/2002 and Q2/2022 were screened. **B** Localization of macrovascular tumour invasion and degree of vessel occlusion. *Abbreviations: CTP Child-Turcotte-Pugh score; FU follow-up; MVI macrovascular tumour invasion*

Overall, 508 consecutive patients with HCC and suspected macrovascular tumour invasion were screened for study inclusion from Q4/2002 until Q2/2022 at the study centre (Supplementary Figure 1). After applying in- and exclusion criteria, 124 patients were finally included in this study (Fig. 1A).

Mean age was 65 ± 10 years and most patients were male (*n* = 110, 89%). The main aetiologies of liver disease were alcohol-related liver disease (*n* = 51, 41%) and viral hepatitis (*n* = 46, 37%), and most patients had liver cirrhosis (*n* = 110, 89%). Of 80 patients (65%) with known variceal status, 50 patients (63%) had gastroesophageal varices (low-risk *n* = 18, 36%; high-risk *n* = 32, 64%). According to the study inclusion criteria, all patients had preserved liver function (CTP class A-B7), with a mean Albumin-to-Bilirubin score (ALBI) of − 2.5 ± 0.4 (stage 1: *n* = 48, 39%; stage 2: *n* = 76, 61%). Overall, 21 patients (17%) had prior surgery/local therapies, 36 individuals had extrahepatic metastases (29%), and more than half of patients had an Eastern Cooperative Oncology Group Performance Status (ECOG-PS) of 0 (*n* = 72, 58%). Baseline radiological assessment was performed in 96 patients (77%) by a CT and in 28 (23%) by an MRI scan. Detailed patient characteristics are displayed in Table 1.

**Table 1:** Baseline patient characteristics

*Patient characteristics*	Study cohort, *n* = 124
Age, years, mean ± SD	65.3 ± 9.7
Sex, n (%)	
Male	110 (89%)
Female	14 (11%)
Aetiology, n (%)	
ARLD	51 (41%)
Viral	46 (37%)
Other/Unknown	15 (12%)
MASLD	12 (10%)
Cirrhosis, n (%)	110 (89%)
Varices, n (%)^a^	50/80 (63%)
Low-risk varices^b^	18/50 (36%)
High-risk varices	32/50 (64%)
Non-selective beta blocker treatment, n (%)	42 (34%)
Endoscopic treatment of varices, n (%)	14 (11%)
CTP score, points, median (IQR)	5 (5–6)
A, n (%)	108 (87%)
B7, n (%)	16 (13%)
ALBI score, mean ± SD	−2.5 ± 0.4
Stage 1, n (%)	48 (39%)
Stage 2, n (%)	76 (61%)
Prior surgery/local therapy, n (%)	21 (17%)
Extrahepatic spread, n (%)	36 (29%)
ECOG PS, n (%)	
0	72 (58%)
1	52 (42%)
Baseline imaging modality, n (%)	
Computed tomography	96 (77%)
Magnetic resonance imaging	28 (23%)

*Abbreviations: ALBI* score albumin-to-bilirubin score, *ARLD* alcohol-related liver disease, *BCLC* Barcelona Clinic Liver Cancer, *BMI* body mass index, *CTP* Child-Turcotte-Pugh score, *ECOG PS* Eastern Cooperative Oncology Group Performance Status, *IQR* interquartile range, MASLD metabolic-dysfunction associated steatotic liver disease, *SD* standard deviation

^a^Data available in 80 patients (65%)

^b^One patient with non-cirrhotic portal hypertension with low-risk varices

### Localization of macrovascular tumour thrombosis at diagnosis

The main portal vein was involved in 47 patients (38%). Invasion of the left and/or right portal branch alone was diagnosed in 53 patients (43%), and 17 individuals (14%) had additional involvement of other splanchnic veins (i.e., hepatic veins, splenic vein, vena cava inferior, superior mesenteric vein). Selective invasion of splanchnic veins without invasion of the main portal vein or its right and left branches was seen in 7 patients (6%) (Table 2 and Fig. 1B). Forty-nine subjects (40%) had non-tumorous thrombus apposition. The number of patients with main portal vein involvement was higher in patients receiving therapeutic doses of anticoagulation compared to all other patients (*n* = 14, 58% vs. *n* = 33, 33%; *p* = 0.022) (Table 2).

**Table 2:** Localization of macrovascular tumour invasion (*n* = 124) and changes during follow-up (*n* = 83) according to therapeutic anticoagulation

*Patient characteristics*	**Study cohort,** ***n*** **= 124 (100%)**	Anticoagulation, ***n*** **= 24 (19%)**	No anticoagulation, ***n*** **= 100 (81%)**	***p*-value**
Localization and degree of occlusion, n (%)				
Main portal vein involved	47 (38%)	14 (58%)	33 (33%)	**0.022**
Total	19 (15%)	6 (25%)	13 (13%)	0.069
Partial	28 (23%)	8 (33%)	20 (20%)	
Left and/or right portal branch	53 (43%)	8 (33%)	45 (45%)	0.299
Total	25 (20%)	2 (8%)	23 (23%)	0.272
Partial	28 (23%)	6 (25%)	22 (22%)	
Left and/or right portal branch and other veins (hepatic veins, splenic vein, VCI, SMV)	17 (14%)	2 (8%)	15 (15%)	0.522
Total	7 (6%)	2 (8%)	5 (5%)	0.237
Partial	10 (8%)	–	10 (10%)	
Other veins (hepatic veins, splenic vein, VCI, SMV)	7 (6%)	–	7 (7%)	0.344
Total	7 (6%)	–	7 (7%)	
Apposition thrombus	49 (40%)	12 (50%)	37 (37%)	0.242
Venous congestion	22 (18%)	5 (21%)	17 (17%)	0.766
*Best radiological response of macrovascular tumour invasion at 3–6 months, n (%)*	**All patients with available FU imaging,** ***n*** **= 83 (100%)**	Anticoagulation, ***n*** **= 17 (20%)**	No anticoagulation, ***n*** **= 66 (80%)**	***p*-value**
Regression	14 (17%)	3 (18%)	11 (17%)	0.951
Stabilization	32 (39%)	7 (41%)	25 (38%)	
Progression	37 (45%)	7 (41%)	30 (45%)	

*Abbreviations: FU* follow-up, *SMV* superior mesenteric vein, *VCI vena cava inferior*

### Description of systemic anti-tumour therapy and anticoagulation

Median estimated follow-up time was 59.0 months (95% confidence interval [95%CI]: 20.2–97.9) and median overall survival was 9.7 months (95%CI: 6.9–12.6). Ninety-eight patients (79%) died during follow-up. Ninety-four patients (76%) were treated with effective systemic therapies, and 19 (15%) and 11 (9%) individuals received either experimental systemic therapy or no systemic therapy, respectively. Most common systemic first-line therapy was sorafenib (*n* = 72, 78%) followed by ICI-based therapy (*n* = 14, 15%). Detailed information on systemic first- and further line treatments are displayed in Supplementary Table 1.

Median time from diagnosis of macrovascular tumour invasion to effective systemic therapy was 1.4 months (95%CI: 1.1–1.7), and median time on effective systemic therapy was 7.9 months (95%CI: 4.5–11.2). Thirty-two patients (26%) were treated with anticoagulation (*n* = 24 with therapeutic and *n* = 8 with reduced/prophylactic doses). Anticoagulation was started in most patients right after diagnosis of macrovascular tumour invasion and some patients were already on anticoagulation for other indications. Median time on therapeutic anticoagulation was 7.7 months (95%CI: 2.2–13.2). Of patients receiving therapeutic doses of anticoagulation, 2 patients (8%) were treated with low molecular weight heparin (LMWH), 5 patients (21%) were treated with vitamin K antagonists (VKA), and 17 patients (71%) received direct oral anticoagulants (DOACs, rivaroxaban *n* = 6, edoxaban *n* = 1, apixaban *n* = 10). Of all 24 patients receiving therapeutic anticoagulation, 12 (50%) were anticoagulated because of an existing non-tumorous thrombus apposition, 3 (13%) individuals without non-tumorous thrombus apposition at baseline received anticoagulation to prevent thrombus apposition, and 9 (38%) patients had another indication for anticoagulation (e.g., atrial fibrillation, history of pulmonary embolism/deep vein thrombosis).

### Impact of systemic anti-tumour therapy and therapeutic anticoagulation on all-cause mortality

**Fig. 2:**
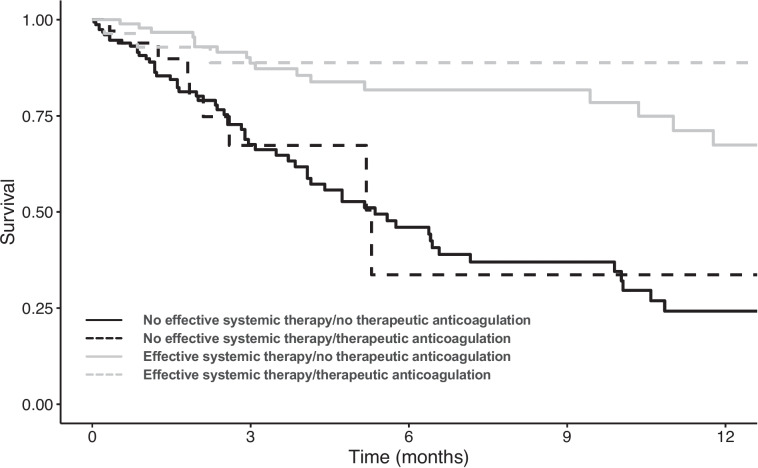
Survival curves for anticoagulation and systemic therapy (time-dependent covariates) using the Simon and Makuch method

In univariable Cox regression analysis, effective systemic therapy (HR: 0.26 [95%CI: 0.16–0.41]; *p* < 0.001) but not therapeutic anticoagulation (HR: 0.71 [95%CI: 0.42–1.19]; *p* = 0.190) was associated with significantly reduced all-cause mortality (Table 3). After adjusting for possible confounding co-factors, effective systemic therapy remained an independent predictor of reduced all-cause mortality (adjusted HR [aHR]: 0.26 [95%CI: 0.16–0.40]; p < 0.001), along with ECOG PS, ALBI score and degree of thrombus-induced vessel occlusion (Table 3). Survival curves according to systemic anti-tumour therapy and anticoagulation status using the Simon and Makuch method are shown in Fig. 2. Importantly, results were similar when including any anticoagulation (therapeutic and reduced/prophylactic doses) in Cox regression analyses (Supplementary Table 2).

**Table 3:** Uni- and multivariable Cox regression analyses of factors associated with all-cause mortality using backward elimination in all patients (*n* = 124, *n* = 98 events)

*Patient characteristics*	Univariable		Multivariable first step		Multivariable last step	
	HR (95%CI)	*p*-value	aHR (95%CI)	*p*-value	aHR (95%CI)	*p*-value
Age, per year	1.00 (0.98–1.03)	0.767	1.00 (0.98–1.02)	1.000	–	–
Sex, male vs. female	1.03 (0.54–1.97)	0.931	0.99 (0.51–1.91)	0.969	–	–
Cirrhosis	1.52 (0.84–2.73)	0.164	1.13 (0.58–2.23)	0.714	–	–
EHS	1.69 (1.17–2.43)	**0.005**	1.21 (0.82–1.79)	0.328	–	–
ECOG PS, 1 vs. 0	1.79 (1.26–2.55)	**0.001**	1.66 (1.15–2.40)	**0.007**	1.73 (1.24–2.42)	**0.001**
CTP score, per point	1.41 (1.09–1.84)	**0.010**	–	–	–	–
ALBI score, per point	2.15 (1.40–3.30)	**< 0.001**	1.82 (1.11–2.98)	**0.017**	2.04 (1.30–3.21)	**0.035**
Degree of thrombus-induced vessel occlusion						
Partial	1	–	1	–	1	–
Total	1.60 (1.11–2.31)	**0.012**	1.47 (1.04–2.07)	**0.027**	1.44 (1.03–2.02)	**0.035**
Thrombus localization						
MPV not involved	1	–	1	–	–	–
MPV involved	0.96 (0.66–1.40)	0.824	1.26 (0.87–1.82)	0.214	–	–
Effective systemic therapy	0.26 (0.16–0.41)	**< 0.001**	0.26 (0.16–0.41)	**< 0.001**	0.26 (0.16–0.40)	**< 0.001**
Non-selective beta blocker therapy	0.81 (0.57–1.14)	0.227	0.71 (0.50–1.00)	0.052	0.75 (0.54–1.05)	0.098
Therapeutic anticoagulation	0.71 (0.42–1.19)	0.190	0.88 (0.55–1.41)	0.596	–	–

*Abbreviations: ALBI* albumin-to-bilirubin score, *(a)HR* (adjusted) hazard ratio, *CTP* Child-Turcotte-Pugh score, *ECOG PS* Eastern Cooperative Oncology Group Performance Status, *EHS* extrahepatic spread, *MPV main portal vein*

### Changes of macrovascular tumour thrombosis during follow-up

Best response of the thrombus at 3–6 months (median time to best response, 3.4 months [95%CI: 3.1–3.7]) was evaluated in patients who had at least one follow-up imaging (*n* = 83, 66%). Of these, 17 patients (20%) received therapeutic anticoagulation (Table 2). There was no difference in the rate of regression (*n* = 3, 18% vs. *n* = 11, 17%), stabilization (*n* = 7, 41% vs. *n* = 25, 38%), and progression (*n* = 7, 41% vs. *n* = 30, 45%) between patients with and without therapeutic anticoagulation (*p* = 0.951) (Table 2, Supplementary Figure 2A).

Similar results were observed, when only patients with non-tumorous thrombus apposition at baseline were analysed (*p* = 0.772), even though the percentage of patients with a thrombus regression was numerically higher in individuals receiving anticoagulation (*n* = 2/7, 29% vs. *n* = 5/26, 19%) (Supplementary Figure 2B).

### Management of varices and portal hypertension-related complications

There was no difference in the number of portal-hypertension-related complications between patients with and without therapeutic anticoagulation, and particularly the proportion of patients with variceal bleeding events was not different between both groups (*n* = 3, 13% vs. *n* = 13, 13%) (Supplementary Table 3). The variceal status was known in 80 patients (65%). This number was higher in patients receiving therapeutic anticoagulation (*n* = 21, 88% vs. *n* = 59, 59%), as was the number of individuals receiving adequate management of varices among those with known variceal status (*n* = 21/21, 100% vs. *n* = 48/59, 81%) (Supplementary Table 3).

Overall, sixty-nine individuals (86%) received adequate management of varices. Variceal bleeding events were observed significantly more often in patients without vs. with adequate management of varices (*n* = 5, 46% vs. *n* = 8, 12%; *p* = 0.014), while there was no difference between both groups regarding other portal hypertension-related complications (Supplemental Table 4). In multivariable logistic regression analysis, adequate management of varices was independently associated with a reduced risk of variceal bleeding (aHR: 0.12 [95%CI: 0.02–0.71]; *p* = 0.019), while no association was observed with degree of thrombus-induced vessel occlusion, involvement of main portal vein, and therapeutic anticoagulation (Table 4). Adequate management of varices was associated with reduced risk of variceal bleeding or death from any cause in univariable Cox regression analysis (HR: 0.46 [95%CI: 0.25–0.84]; *p* = 0.011) but not in multivariable analyses (Supplementary Table 5). However, when only including patients with involvement of the main portal vein and/or both portal branches who have the highest bleeding risk, adequate management of varices was independently associated with reduced risk of variceal bleeding or death from any cause (aHR: 0.29 [95%CI: 0.13–0.66]; *p* = 0.003) (Supplementary Table 6).

**Table 4:** Uni- and multivariable logistic regression analyses of factors associated with variceal bleeding using backward elimination in patients with known variceal status at study inclusion (*n* = 80, *n* = 13 events)

*Patient characteristics*	Univariable		Multivariable first step		Multivariable last step	
	HR (95%CI)	p-value	aHR (95%CI)	p-value	aHR (95%CI)	p-value
Age, per year	0.90 (0.84–0.98)	**0.008**	0.88 (0.80–0.97)	**0.013**	0.90 (0.82–0.98)	**0.022**
EHS	0.37 (0.08–1.83)	0.223	0.09 (0.01–1.14)	0.063	0.20 (0.03–1.22)	0.081
ECOG PS, 1 vs. 0	2.23 (0.67–7.42)	0.190	9.02 (1.36–59.87)	**0.023**	5.91 (1.22–28.73)	**0.028**
CTP score, per point	0.64 (0.26–1.55)	0.323	–	–	–	–
ALBI score, per point	1.33 (0.34–5.29)	0.685	0.31 (0.03–2.94)	0.308	–	–
Degree of thrombus-induced vessel occlusion						
Partial	1	–	1	–	–	–
Total	1.07 (0.32–3.51)	0.915	2.79 (0.45–17.44)	0.274	–	–
Thrombus localization						
MPV not involved	1	–	1	–	–	–
MPV involved	0.58 (0.18–1.91)	0.369	3.44 (0.59–20.09)	0.171	–	–
Effective systemic therapy	0.54 (0.14–2.04)	0.364	0.36 (0.04–3.04)	0.347	–	–
Adequate management of varices	0.16 (0.04–0.64)	**0.009**	0.03 (0.00–0.38)	**0.006**	0.12 (0.02–0.71)	**0.019**
Therapeutic anticoagulation	0.82 (0.20–3.31)	0.777	1.73 (0.26–11.62)	0.574	–	–

*Abbreviations: ALBI* albumin-to-bilirubin score, *(a)HR* (adjusted) hazard ratio, *CTP* Child-Turcotte-Pugh score, *ECOG PS* Eastern Cooperative Oncology Group Performance Status, *EHS* extrahepatic spread, *MPV* main portal vein

In the whole cohort (*n* = 124), the use of NSBB was independently associated with reduced risk of variceal bleeding or death from any cause (aHR: 0.69 [95%CI: 0.50–0.96]; *p* = 0.027), along with effective anti-tumour therapy, partial vessel occlusion, lower ALBI score, and better performance status (Supplementary Table 7).

## Discussion

In this retrospective study including 124 patients with HCC and macrovascular tumour invasion, therapeutic anticoagulation was not associated with an increased bleeding rate but failed to reduce thrombosis progression and mortality risk. Adequate management of varices was associated with a lower rate and risk of variceal bleedings, especially in patients with involvement of the main portal vein and/or both portal branches, where it also reduced the risk of variceal bleeding or death from any cause.

In patients with cirrhosis and non-tumorous portal vein thrombosis, therapeutic anticoagulation is recommended in candidates for liver transplantation as well as in selected non-transplant candidates (i.e., recent > 50% occlusion of main portal vein or both main branches or involvement of mesenteric veins), with the aim to ‘recanalize’ the portal venous tract to prevent complications or facilitate liver transplantation. Evaluation of treatment efficacy by imaging is recommended after 3–6 months [7, 18, 27].

No recommendations on anticoagulation exist for patients with HCC and macrovascular tumour invasion. Regression of a tumour thrombus, as seen with systemic anti-tumour therapy in some cases [28], seems unlikely to be achievable by anticoagulation. However, anticoagulation may prevent occurrence or progression of non-malignant thrombus apposition that could worsen portal hypertension or cause thromboembolic complications, providing a clinical rationale for the use of anticoagulation in this setting.

In patients with at least one follow-up imaging, the regression rate at 3–6 months was similar between patients with and without therapeutic anticoagulation (18% vs. 17%), as was the rate of progression (41% vs. 45%). Results were similar when only patients with thrombus apposition at baseline were analysed. Not surprisingly, this contrasts with cirrhotic patients with non-tumorous portal vein thrombosis, in whom the ‘recanalization rate’ was significantly higher with anticoagulation; however, the progression rate in our study was comparable to that observed in cirrhotic individuals with untreated portal vein thrombosis [29–31].

The rate of portal hypertension-related complications was also similar between patients with and without therapeutic anticoagulation. In particular, we did not observe a reduced or increased number of variceal bleeding events in patients receiving anticoagulation, which is in line with previous reports of cirrhotic patients receiving anticoagulation [31–33].

Like non-tumorous portal vein thrombosis, macrovascular tumour invasion, especially in case of main portal vein involvement, may aggravate portal hypertension by increasing resistance to portal blood flow [7, 12]. In line, portal vein tumour thrombosis is associated with an increased risk of high-risk varices and variceal bleeding in patients with HCC, particularly in individuals receiving vascular endothelial growth factor (VEGF)-targeted agents [13]. Although the management of portal hypertension in cirrhotic patients with HCC should follow recommendations for individuals with liver cirrhosis, there are several uncertainties, including the value of NSBBs in patients with varices secondary to portal vein tumour thrombosis [12, 15–17].

In our study, variceal status was known in two-thirds of patients (anticoagulation vs. no anticoagulation, 88% vs. 59%). This is higher compared to two previous reports on advanced HCC patients treated with atezolizumab plus bevacizumab (53% in both) [34]. In patients with known variceal status, 86% of individuals received adequate bleeding prophylaxis in our cohort. This proportion was higher in patients receiving anticoagulation (100%) than in those without anticoagulation (81%), suggesting that treating physicians were more cautious and diligent in adhering to guidelines when initiating anticoagulation. Overall, these data call for measures to raise the awareness for adequate screening and management of portal hypertension in patients with HCC.

Only little is known about the efficacy of bleeding prophylaxis in patients with HCC. In a large Korean cohort of HCC patients without a history of variceal bleeding, primary prophylaxis was associated with a lower cumulative incidence rate of variceal haemorrhage at one year; however, only overall mortality but not the risk of variceal bleeding was significantly reduced with primary prophylaxis [15, 35]. In our cohort, the number of variceal bleeding events was significantly lower in individuals with adequate management of varices, as was the risk for variceal bleeding or death from any cause in patients with involvement of the main portal vein and/or both portal branches after multivariable adjustment.

We want to acknowledge some limitations of our study. These include the retrospective nature with all its inevitable, potential confounders. To account for a potential selection bias due to lack of randomisation, main analyses were adjusted for relevant co-factors in multivariable models. Furthermore, the variceal status was only known in 80 of 124 patients; therefore, only these were available to evaluate the efficacy of variceal management. Different types of anticoagulation (i.e., LMWH, VKA, DOAC) were used in our cohort, but the sample size was not large enough to analyse the effects of each type separately. Finally, follow-up imaging in patients with advanced HCC is usually performed every 3 months at our institution; nevertheless, imaging was done by different modalities (i.e., CT or MRI) and not performed at predefined intervals based on a specific protocol.

## Conclusion

In conclusion, therapeutic anticoagulation had no clinical benefit in patients with HCC and macrovascular tumour invasion, but adequate management of varices (i.e., NSBB and/or endoscopic treatment of varices) was associated with reduced risk of variceal bleeding events. Hence, our data support the use of variceal bleeding prophylaxis as recommended for patients with liver cirrhosis in HCC patients with macrovascular tumour invasion, but do not argue for therapeutic anticoagulation in this setting. Prospective trials are warranted to confirm these findings.

### Supplementary Information

**Additional file 1: Supplementary Figure 1. **Illustration of inclusion and follow-up time. **Supplementary Figure 2.** Changes of macrovascular tumour invasion during follow-up. **Supplementary Table 1.** Description of systemic anti-tumour therapy and anticoagulation. **Supplementary Table 2.** Uni- and multivariable Cox regression analyses of factors associated with all-cause mortality using backward elimination considering all types of anticoagulation in all patients (*n*=124, *n*=98 events). **Supplementary Table 3.** Management of varices and portal hypertension-related complications according to therapeutic anticoagulation status. **Supplementary Table 4.** Decompensation events according to adequate management of varices. **Supplementary Table 5.** Uni- and multivariable Cox regression analyses of factors associated with risk of variceal bleeding or death from any-cause using backward elimination in patients with known variceal status at study inclusion (*n*=80, *n*=61 events). **Supplementary Table 6.** Uni- and multivariable Cox regression analyses of factors associated with risk of variceal bleeding or death from any cause using backward elimination in patients with known variceal status and involvement of the main portal vein and/or both portal branches at study inclusion (*n*=63, *n*=35 events). **Supplementary Table 7.** Uni- and multivariable Cox regression analyses of factors associated with risk of variceal bleeding or death from any-cause using backward elimination in all patients (*n*=124, *n*=99 events).

## Data Availability

The data that support the findings of this study are available from the corresponding author upon reasonable request.

## Abbreviations

(a)HR: (adjusted) hazard ratio
ALBI: Albumin-to-Bilirubin score
BCLC: Barcelona-Clinic Liver Cancer
CI: confidence interval
CT: computed tomography
CTP: Child-Turcotte-Pugh score
DOAC: direct oral anticoagulants
ECOG-PS: Eastern Cooperative Oncology Group Performance Status
HCC: hepatocellular carcinoma
ICI: immune checkpoint inhibitor
IQR: interquartile range
LI-RADS: Liver Imaging and Data System
LMWH: low molecular weight heparin
MRI: magnetic resonance imaging
MVI: macrovascular tumour invasion
NSBB: non-selective beta blocker
SD: standard deviation
SIRT: selective internal radiation therapy
TACE: transarterial chemoembolization
TKI: tyrosine kinase inhibitors
VEGF: vascular endothelial growth factor
VKA: vitamin K antagonist
