# Supplementary material for: Management of varices but not anticoagulation is associated with improved outcome in patients with HCC and macrovascular tumour invasion
Source: Cancer Imaging. 2024 Jan 13;24:9. doi: 10.1186/s40644-024-00657-z (PMC10787425; doi:10.1186/s40644-024-00657-z)
Supplement: Supplementary file 1 — Additional file 1: Supplementary Figure 1. Illustration of inclusion and follow-up time. Supplementary Figure 2. Changes of macrovascular tumour invasion during follow-up. Supplementary Table 1. Description of systemic anti-tumour therapy and anticoagulation. Supplementary Table 2. Uni- and multivariable Cox regression analyses of factors associated with all-cause mortality using backward elimination considering all types of anticoagulation in all patients (n=124, n=98 events). Supplementary Table 3. Management of varices and portal hypertension-related complications according to therapeutic anticoagulation status. Supplementary Table 4. Decompensation events according to adequate management of varices. Supplementary Table 5. Uni- and multivariable Cox regression analyses of factors associated with risk of variceal bleeding or death from any-cause using backward elimination in patients with known variceal status at study inclusion (n=80, n=61 events). Supplementary Table 6. Uni- and multivariable Cox regression analyses of factors associated with risk of variceal bleeding or death from any cause using backward elimination in patients with known variceal status and involvement of the main portal vein and/or both portal branches at study inclusion (n=63, n=35 events). Supplementary Table 7. Uni- and multivariable Cox regression analyses of factors associated with risk of variceal bleeding or death from any-cause using backward elimination in all patients (n=124, n=99 events). [file 40644_2024_657_MOESM1_ESM.docx]

***Management of varices but not anticoagulation is associated with improved outcome in patients with hepatocellular carcinoma and macrovascular tumour invasion***

**SUPPLEMENTARY MATERIALS**

**Lorenz Balcar M.D. ^1,2^, Arpad Mrekva M.D. ^3^**, Bernhard Scheiner M.D. Ph.D. ^1,2^, Katharina Pomej M.D. ^1,2^, Tobias Meischl M.D. ^1,2,4^, Mattias Mandorfer M.D. Ph.D. ^1^, Thomas Reiberger M.D. ^1^, Michael Trauner M.D. ^1^, **Dietmar Tamandl M.D. ^3^, Matthias Pinter M.D. Ph.D. ^1,2^**

**Author names in bold designate shared first (L.B. and A.M.) and last (D.T. and M.P.) authorship.**

1 Division of Gastroenterology and Hepatology, Department of Internal Medicine III, Medical University of Vienna, Vienna, Austria

2 Liver Cancer (HCC) Study Group Vienna, Medical University of Vienna, Vienna, Austria

3 Department of Biomedical Imaging and Image-Guided Therapy, Medical University of Vienna, Vienna, Austria

4 3^rd^ Medical Department (Haematology & Oncology), Hanusch Krankenhaus, Vienna

**Correspondence:** Matthias Pinter, M.D. Ph.D.

Division of Gastroenterology and Hepatology

Department of Internal Medicine III

Medical University of Vienna

Waehringer Guertel 18-20, 1090 Vienna, Austria

P: +43 1 40400 47440, F: +43 1 40400 47350

M: matthias.pinter@meduniwien.ac.at

**TABLE OF CONTENTS**

[SUPPLEMENTARY FIGURES 3](#_Toc125915775)

[Supplementary Figure 1 3](#_Toc125915776)

[Supplementary Figure 2A 4](#_Toc125915777)

[Supplementary Figure 2B 5](#_Toc125915778)

[SUPPLEMENTARY TABLES 7](#_Toc125915779)

[Supplementary Table 1 7](#_Toc125915780)

[Supplementary Table 2 9](#_Toc125915781)

[Supplementary Table 3 10](#_Toc125915782)

[Supplementary Table 4 11](#_Toc125915783)

[Supplementary Table 5 12](#_Toc125915784)

[Supplementary Table 6 13](#_Toc125915785)

[Supplementary Table 7 14](#_Toc125915786)

# SUPPLEMENTARY FIGURES

## Supplementary Figure 1

**Supplementary Figure 1.** Illustration of inclusion and follow-up time.

## Supplementary Figure 2A

## Supplementary Figure 2B

**Supplementary Figure 2. Changes of macrovascular tumour invasion during follow-up. (A)** Best radiological response of macrovascular tumour invasion at 3-6 months in patients with available follow-up imaging. **(B)** Best radiological response of macrovascular tumour invasion at 3-6 months in patients with non-tumorous thrombus apposition at baseline and available follow-up imaging.

*Abbreviations: AC anticoagulation, MVI macrovascular tumour invasion*

# SUPPLEMENTARY TABLES

## Supplementary Table 1

| *Patient characteristics* | | | | **Study cohort,**  **n=124** |
| --- | --- | --- | --- | --- |
| Systemic therapy, n (%) | | | | |
|  | None | | | 11 (9%) |
|  | Experimental, n (%) | | | 19 (15%) |
|  | Effective, n (%) | | | 94 (76%) |
|  | Type of effective systemic 1^st^ line therapy | | | |
|  |  | Sorafenib | | 72 (78%) |
|  |  | ICI-based therapy | | 14 (15%) |
|  |  | Cabozantinib | | 1 (1%) |
|  |  | Lenvatinib | | 1 (1%) |
|  | Type of effective systemic 2^nd^ line therapy | | | |
|  |  | Regorafenib | | 9 (10%) |
|  |  | ICI-based therapy | | 5 (5%) |
|  |  | Sorafenib | | 5 (5%) |
|  |  | Lenvatinib | | 5 (5%) |
|  |  | Cabozantinib | | 1 (1%) |
|  | Type of effective 3^rd^ line systemic therapy | | | |
|  |  | Cabozantinib | | 5 (5%) |
|  |  | ICI-based therapy | | 3 (3%) |
|  |  | Regorafenib | | 3 (3%) |
|  |  | Lenvatinib | | 2 (2%) |
|  |  | Sorafenib | | 1 (1%) |
|  | Type of effective further lines systemic therapy | | | |
|  |  | Cabozantinib | | 3 (3%) |
|  |  | Regorafenib | | 1 (1%) |
|  |  | Lenvatinib | | 1 (1%) |
|  | Median time from MVI diagnosis to effective systemic therapy (months), median (95% CI) | | | 1.4 (1.1-1.7) |
|  | Median time on effective systemic therapy (months), median (95%CI) | | | 7.9 (4.5-11.2) |
| Anticoagulation therapy, n (%) | | | | 32 (26%) |
|  | Reduced/prophylactic dose, n (%) | | | 8 (6%) |
|  | Therapeutic dose, n (%) | | | 24 (19%) |
|  | Type of anticoagulation, n (%) | | | |
|  | Reduced/prophylactic dose | | | 8 (100%) |
|  |  | LMWH | | 6 (75%) |
|  |  | Edoxaban | | 2 (25%) |
|  | Therapeutic dose | | | 24 (100%) |
|  |  | LMWH | | 2 (8%) |
|  |  | VKA | | 5 (21%) |
|  |  | DOAC | | 17 (71%) |
|  |  |  | Rivaroxaban | 6 (25%) |
|  |  |  | Edoxaban | 1 (4%) |
|  |  |  | Apixaban | 10 (42%) |
|  | Median time from MVI diagnosis to therapeutic anticoagulation (months), median (95%CI) | | | 0 (0-2.0) |
|  | Median time on therapeutic anticoagulation (months), median (95%CI) | | | 7.7 (2.2-13.2) |

**Supplementary Table 1.** Description of systemic anti-tumour therapy and anticoagulation.

*Abbreviations: CI confidence interval; DOAC direct oral anticoagulant; ICI immune checkpoint inhibitor; LMWH low molecular weight heparin; MVI macrovascular tumour invasion; VKA vitamin K antagonist*

## Supplementary Table 2

| *Patient characteristics* | | **Univariable** | | **Multivariable**  **first step** | | **Multivariable**  **last step** | |
| --- | --- | --- | --- | --- | --- | --- | --- |
|  |  | **HR (95%CI)** | **p-value** | **aHR (95%CI)** | **p-value** | **aHR (95%CI)** | **p-value** |
| Age, per year | | 1.00 (0.98-1.03) | 0.767 | 1.00 (0.98-1.02) | 0.941 | - | - |
| Sex, male vs. female | | 1.03 (0.54-1.97) | 0.931 | 0.99 (0.51-1.91) | 0.981 | - | - |
| Cirrhosis | | 1.52 (0.84-2.73) | 0.164 | 1.15 (0.58-2.26) | 0.697 | - | - |
| EHS | | 1.69 (1.17-2.43) | **0.005** | 1.20 (0.81-1.76) | 0.361 | - | - |
| ECOG PS, 1 vs. 0 | | 1.79 (1.26-2.55) | **0.001** | 1.66 (1.15-2.39) | **0.006** | 1.71 (1.22-2.40) | **0.002** |
| CTP score, per point | | 1.41 (1.09-1.84) | **0.010** | - | - | - | - |
| ALBI score, per point | | 2.15 (1.40-3.30) | **<0.001** | 1.84 (1.13-3.00) | **0.014** | 2.05 (1.31-3.23) | **0.002** |
| Degree of thrombus-induced vessel occlusion | | | | | | | |
|  | Partial | 1 | - | 1 | - | 1 | - |
|  | Total | 1.60 (1.11-2.31) | **0.012** | 1.46 (1.03-2.06) | **0.032** | 1.42 (1.01-2.00) | **0.044** |
| Thrombus localization | | | | | | | |
|  | MPV not involved | 1 | - | 1 | - | - | - |
|  | MPV involved | 0.96 (0.66-1.40) | 0.824 | 1.25 (0.88-1.77) | 0.223 | - | - |
| Effective systemic therapy | | 0.27 (0.17-0.41) | **<0.001** | 0.27 (0.17-0.42) | **<0.001** | 0.27 (0.17-0.41) | **<0.001** |
| Non-selective beta blocker therapy | | 0.81 (0.57-1.14) | 0.227 | 0.73 (0.52-1.02) | 0.068 | 0.76 (0.55-1.07) | 0.113 |
| Any Anticoagulation | | 0.79 (0.51-1.21) | 0.269 | 0.92 (0.60-1.40) | 0.689 | - | - |

**Supplementary Table 2.** Uni- and multivariable Cox regression analyses of factors associated with all-cause mortality using backward elimination considering all types of anticoagulation in all patients (n=124, n=98 events).

*Abbreviations: ALBI albumin-to-bilirubin score; (a)HR (adjusted) hazard ratio; CTP Child-Turcotte-Pugh score; ECOG PS Eastern Cooperative Oncology Group Performance Status; EHS extrahepatic spread; MPV main portal vein*

## Supplementary Table 3

| *Patient characteristics* | | **Study cohort,**  **n=124 (100%)** | **Anticoagulation,**  **n=24 (19%)** | **No anticoagulation,**  **n=100 (81%)** | **p-value** |
| --- | --- | --- | --- | --- | --- |
| *Management of varices* | | | | | |
|  | Prior bleeding, n (%) | 10 (8%) | - | 10 (10%) | 0.207 |
|  | Non-selective beta blocker therapy during follow-up, n (%) | 51 (41%) | 11 (46%) | 40 (40%) | 0.602 |
|  | Median time on non-selective beta blocker therapy during follow-up, median (95%CI) | 10.7 (6.1-15.2) | 7.4 (6.3-8.4) | 10.9 (8.4-13.5) | 0.210 |
|  | Adequate management of varices, n (%)^1^ | 69 (86%) | 21 (100%) | 48 (81%) | 0.058 |
| Decompensation events during follow-up, n (%) | | | | | |
|  | Variceal bleeding | 16 (13%) | 3 (13%) | 13 (13%) | 1.000 |
|  | Ascites | 39 (31%) | 10 (42%) | 29 (29%) | 0.230 |
|  | Hepatic encephalopathy | 10 (8%) | - | 10 (10%) | 0.207 |
|  | Hepatorenal syndrome-acute kidney injury | 3 (2%) | - | 3 (3%) | 1.000 |
|  | Spontaneous bacterial peritonitis | 8 (6%) | - | 8 (8%) | 0.352 |
|  | ≥1 decompensation event during follow-up | 51 (41%) | 11 (46%) | 40 (40%) | 0.602 |

^1^ data available in 80 patients (65%)

**Supplementary Table 3.** Management of varices and portal hypertension-related complications according to therapeutic anticoagulation status.

*Abbreviations: CI confidence interval*

## Supplementary Table 4

| *Patient characteristics* | | **Variceal status known,**  **n=80 (100%)** | **Adequate management of varices**  **n=69 (86%)** | **Inadequate management of varices,**  **n=11 (14%)** | **p-value** |
| --- | --- | --- | --- | --- | --- |
| Decompensation events during follow-up, (%) | | | | | |
|  | Variceal bleeding | 13 (16%) | 8 (12%) | 5 (46%) | **0.014** |
|  | Ascites | 27 (34%) | 23 (33%) | 4 (36%) | 1.000 |
|  | Hepatic encephalopathy | 7 (9%) | 6 (9%) | 1 (9%) | 1.000 |
|  | Hepatorenal syndrome-acute kidney injury | 3 (4%) | 3 (4%) | - | 1.000 |
|  | Spontaneous bacterial peritonitis | 5 (6%) | 4 (6%) | 1 (9%) | 0.533 |
|  | Any decompensation event | 35 (44%) | 29 (42%) | 6 (55%) | 0.521 |

**Supplementary Table 4.** Decompensation events according to adequate management of varices.

## Supplementary Table 5

| *Patient characteristics* | | **Univariable** | | **Multivariable**  **first step** | | **Multivariable**  **last step** | |
| --- | --- | --- | --- | --- | --- | --- | --- |
|  |  | **HR (95%CI)** | **p-value** | **aHR (95%CI)** | **p-value** | **aHR (95%CI)** | **p-value** |
| Age, per year | | 1.00 (0.97-1.03) | 0.848 | 0.98 (0.96-1.01) | 0.212 | - | - |
| EHS | | 1.54 (0.97-2.46) | 0.068 | 0.67 (0.38-1.19) | 0.175 | - | - |
| ECOG PS, 1 vs. 0 | | 2.23 (1.36-3.67) | **0.002** | 2.90 (1.65-5.10) | **<0.001** | 2.26 (1.43-3.56) | **<0.001** |
| CTP score, per point | | 1.48 (1.10-2.01) | **0.010** | - | - | - | - |
| ALBI score, per point | | 2.61 (1.61-4.24) | **<0.001** | 1.70 (0.93-3.10) | 0.087 | 1.89 (1.10-3.25) | **0.021** |
| Degree of thrombus-induced vessel occlusion | | | | | | | |
|  | Partial | 1 | - | 1 | - | - | - |
|  | Total | 1.69 (1.04-2.75) | **0.035** | 1.73 (1.05-2.86) | **0.032** | 1.46 (0.94-2.28) | 0.091 |
| Thrombus localization | | | | | | | |
|  | MPV not involved | 1 | - | 1 | - | - | - |
|  | MPV involved | 1.01 (0.62-1.62) | 0.980 | 1.16 (0.73-1.84) | 0.521 | - | - |
| Effective systemic therapy | | 0.24 (0.13-0.41) | **<0.001** | 0.27 (0.15-0.48) | **<0.001** | 0.26 (0.15-0.46) | **<0.001** |
| Adequate management of varices | | 0.46 (0.25-0.84) | **0.011** | 0.54 (0.28-1.07) | 0.076 | - | - |
| Therapeutic anticoagulation | | 0.61 (0.34-1.11) | 0.105 | 0.90 (0.50-1.62) | 0.716 | - | - |

**Supplementary Table 5.** Uni- and multivariable Cox regression analyses of factors associated with risk of variceal bleeding or death from any-cause using backward elimination in patients with known variceal status at study inclusion (n=80, n=61 events).

*Abbreviations: ALBI albumin-to-bilirubin score; (a)HR (adjusted) hazard ratio; CTP Child-Turcotte-Pugh score; ECOG PS Eastern Cooperative Oncology Group Performance Status; EHS extrahepatic spread; MPV main portal vein*

## Supplementary Table 6

| *Patient characteristics* | | **Univariable** | | **Multivariable**  **first step** | | **Multivariable**  **last step** | |
| --- | --- | --- | --- | --- | --- | --- | --- |
|  |  | **HR (95%CI)** | **p-value** | **aHR (95%CI)** | **p-value** | **aHR (95%CI)** | **p-value** |
| Age, per year | | 0.97 (0.93-1.02) | 0.291 | 0.98 (0.94-1.03) | 0.460 | - | - |
| EHS | | 1.75 (0.93-3.28) | 0.081 | 0.69 (0.29-1.65) | 0.403 | - | - |
| ECOG PS, 1 vs. 0 | | 2.31 (1.19-4.48) | **0.013** | 2.98 (1.36-6.54) | **0.007** | 2.19 (1.21-3.94) | **0.009** |
| CTP score, per point | | 1.46 (0.97-2.21) | 0.072 | - | - | - | - |
| ALBI score, per point | | 2.27 (1.14-4.52) | **0.019** | 0.75 (0.25-2.24) | 0.607 | - | - |
| Degree of thrombus-induced vessel occlusion | | | | | | | |
|  | Partial | 1 | - | 1 | - | - | - |
|  | Total | 1.68 (0.83-3.38) | 0.149 | 1.90 (0.88-4.09) | 0.103 | - | - |
| Effective systemic therapy | | 0.17 (0.07-0.40) | **<0.001** | 0.16 (0.06-0.40) | **<0.001** | 0.16 (0.07-0.39) | **<0.001** |
| Adequate management of varices | | 0.33 (0.14-0.75) | **0.009** | 0.33 (0.15-0.74) | **0.007** | 0.29 (0.13-0.66) | **0.003** |
| Therapeutic anticoagulation | | 0.60 (0.29-1.26) | 0.176 | 0.64 (0.31-1.35) | 0.240 | - | - |

**Supplementary Table 6.** Uni- and multivariable Cox regression analyses of factors associated with risk of variceal bleeding or death from any cause using backward elimination in patients with known variceal status and involvement of the main portal vein and/or both portal branches at study inclusion (n=63, n=35 events).

*Abbreviations: ALBI albumin-to-bilirubin score; (a)HR (adjusted) hazard ratio; CTP Child-Turcotte-Pugh score; ECOG PS Eastern Cooperative Oncology Group Performance Status; EHS extrahepatic spread*

## Supplementary Table 7

| *Patient characteristics* | | **Univariable** | | **Multivariable**  **first step** | | **Multivariable**  **last step** | |
| --- | --- | --- | --- | --- | --- | --- | --- |
|  |  | **HR (95%CI)** | **p-value** | **aHR (95%CI)** | **p-value** | **aHR (95%CI)** | **p-value** |
| Age, per year | | 1.00 (0.98-1.02) | 0.986 | 0.99 (0.98-1.01) | 0.575 | - | - |
| Sex, male vs. female | | 1.07 (0.56-2.04) | 0.831 | 1.02 (0.55-1.89) | 0.953 | - | - |
| Cirrhosis | | 1.44 (0.79-2.65) | 0.235 | 1.11 (0.57-2.14) | 0.763 | - | - |
| EHS | | 1.62 (1.12-2.33) | **0.010** | 1.13 (0.76-1.67) | 0.538 | - | - |
| ECOG PS, 1 vs. 0 | | 1.85 (1.29-2.67) | **0.001** | 1.75 (1.20-2.55) | **0.004** | 1.79 (1.28-2.50) | **0.001** |
| CTP score, per point | | 1.42 (1.09-1.84) | **0.008** | - | - | - | - |
| ALBI score, per point | | 2.20 (1.43-3.37) | **<0.001** | 1.84 (1.12-3.01) | **0.016** | 2.02 (1.30-3.14) | **0.002** |
| Degree of thrombus-induced vessel occlusion | | | | | | | |
|  | Partial | 1 | - | 1 | - | 1 | - |
|  | Total | 1.66 (1.13-2.44) | **0.009** | 1.49 (1.05-2.12) | **0.027** | 1.46 (1.03-2.07) | **0.034** |
| Thrombus localization | | | | | | | |
|  | MPV not involved | 1 | - | 1 | - | - | - |
|  | MPV involved | 0.94 (0.64-1.39) | 0.753 | 1.20 (0.82-1.77) | 0.348 | - | - |
| Effective systemic therapy | | 0.32 (0.21-0.50) | **<0.001** | 0.33 (0.21-0.52) | **<0.001** | 0.33 (0.22-0.52) | **<0.001** |
| Non-selective beta blocker therapy | | 0.74 (0.52-1.05) | 0.089 | 0.66 (0.47-0.93) | **0.017** | 0.69 (0.50-0.96) | **0.027** |
| Therapeutic anticoagulation | | 0.68 (0.40-1.16) | 0.160 | 0.88 (0.54-1.43) | 0.612 | - | - |

**Supplementary Table 7**. Uni- and multivariable Cox regression analyses of factors associated with risk of variceal bleeding or death from any-cause using backward elimination in all patients (n=124, n=99 events).

*Abbreviations: ALBI albumin-to-bilirubin score; (a)HR (adjusted) hazard ratio; CTP Child-Turcotte-Pugh score; ECOG PS Eastern Cooperative Oncology Group Performance Status; EHS extrahepatic spread; MPV main portal vein*
